# A sustainable and green HPLC-PDA technique for the simultaneous estimation of Post-COVID-19 syndrome co-administered drugs with greenness and whiteness assessment

**DOI:** 10.1038/s41598-024-75216-4

**Published:** 2024-10-31

**Authors:** Passant M. Medhat, Manal Mohamed Fouad, Hany H. Monir, Nermine S. Ghoniem

**Affiliations:** 1 Analytical Chemistry Department, Faculty of Pharmacy, October University for Modern Sciences and Arts (MSA), 11787 6th October City, Egypt; 2 Analytical Chemistry Department, Faculty of Pharmacy (Girls), Al-Azhar University, Nasr City, Cairo Egypt; 3 Pharmaceutical Analytical Chemistry Department, Faculty of Pharmacy, Cairo University, Kasr El-Aini Street, Cairo, ET-11562 Egypt

**Keywords:** Analytical chemistry, Green chemistry

## Abstract

COVID-19 has been a growing global concern in the past four years. Several syndromes associated with this multi-organ viral infection have been observed since the outbreak. According to estimates, 10–15% of people with SARS-CoV- infection may have post-COVID-19 syndrome. Even months after infection, common residual signs and symptoms include myalgia, exhaustion, shortness of breath, rapid heartbeat, stroke, and memory and cognitive impairment which can negatively affect survivors’ quality of life and may consequently lead to their death. Therefore, it is necessary to think about potential therapy options for dealing with both short and long-term impacts. Paracetamol (a common analgesic and antipyretic) and Dexketoprofen Trometamol (a non-steroidal anti-inflammatory drug) are used together to relieve post-COVID symptoms like myalgia (muscle pain) and headache. Additionally, to prevent thrombotic events, Rivaroxaban is recommended for 35 days following discharge. Thus an eco-friendly HPLC-DAD technique was developed for simultaneous quantification of Paracetamol, Dexketoprofen Trometamol, and Rivaroxaban which are co-administered for treatment of post-COVID-19 syndrome. The suggested method was found to be linear in the concentration ranges of 3.00–45.00 µg/mL, 0.5–50.00 µg/mL, and 0.15–20.00 µg/mL, and a limit of detection down to 0.531 µg/mL, 0.095 µg/mL and 0.047 µg/mL for Paracetamol, Dexketoprofen Trometamol and Rivaroxaban, respectively. This method was effectively used to quantify the studied drugs in their bulk powder and spiked human plasma with high percentage recoveries (96.55–99.46%). The suggested approach was validated per International Conference on Harmonization (ICH) requirements and found to be within the acceptable ranges. The method was developed using Green Analytical Chemistry (GAC) principles, with the solvents used and run time having a significant effect on the method’s greenness. “Non-toxic” ethanol served as the organic modifier in the mobile phase, moreover, the total run time was 12 min making it suitable for the routine analysis of the mentioned drugs in plasma samples. To get a full image of the method’s greenness profile; two most recent greenness assessment tools, the Green Analytical Procedure Index (GAPI), and the Analytical GREEnness metric (AGREE), were employed, with White Analytical Chemistry (WAC) principles proving its environmental safety.

**Supplementary Information:**

The online version contains supplementary material available at 10.1038/s41598-024-75216-4.

## Keywords

Post-COVID syndrome, HPLC-DAD, Paracetamol, Dexketoprofen Trometamol and Rivaroxaban.

## Introduction

COVID-19 pandemic, which was first identified in Wuhan, China, in December 2019, spread quickly to become the largest acute pandemic tragedy in human history^1–4^. 

According to WHO,75 nations reported COVID-19 cases and 43 countries reported COVID-19 deaths during the 28-day period from January 8, 2024, to February 4, 2024. During this period, there were over 119,000 new hospital admissions and over 1500 new ICU admissions recorded. Recent data reported that during the period from (January 29 to February 4, 2024) about 10% of SARS-CoV-2 PCR showed a positive result, which proves that this virus is still life-threatening to date^5^.

Common symptoms of COVID-19 include fever, myalgia, and exhaustion combined with more respiratory symptoms such as sore throat, cough, and shortness of breath. Since the pandemic started, neurological symptoms involving the central and peripheral nervous systems have also been discovered^6–8^. Furthermore, COVID-19 impacts all organs and systems.

**Fig. 1:**
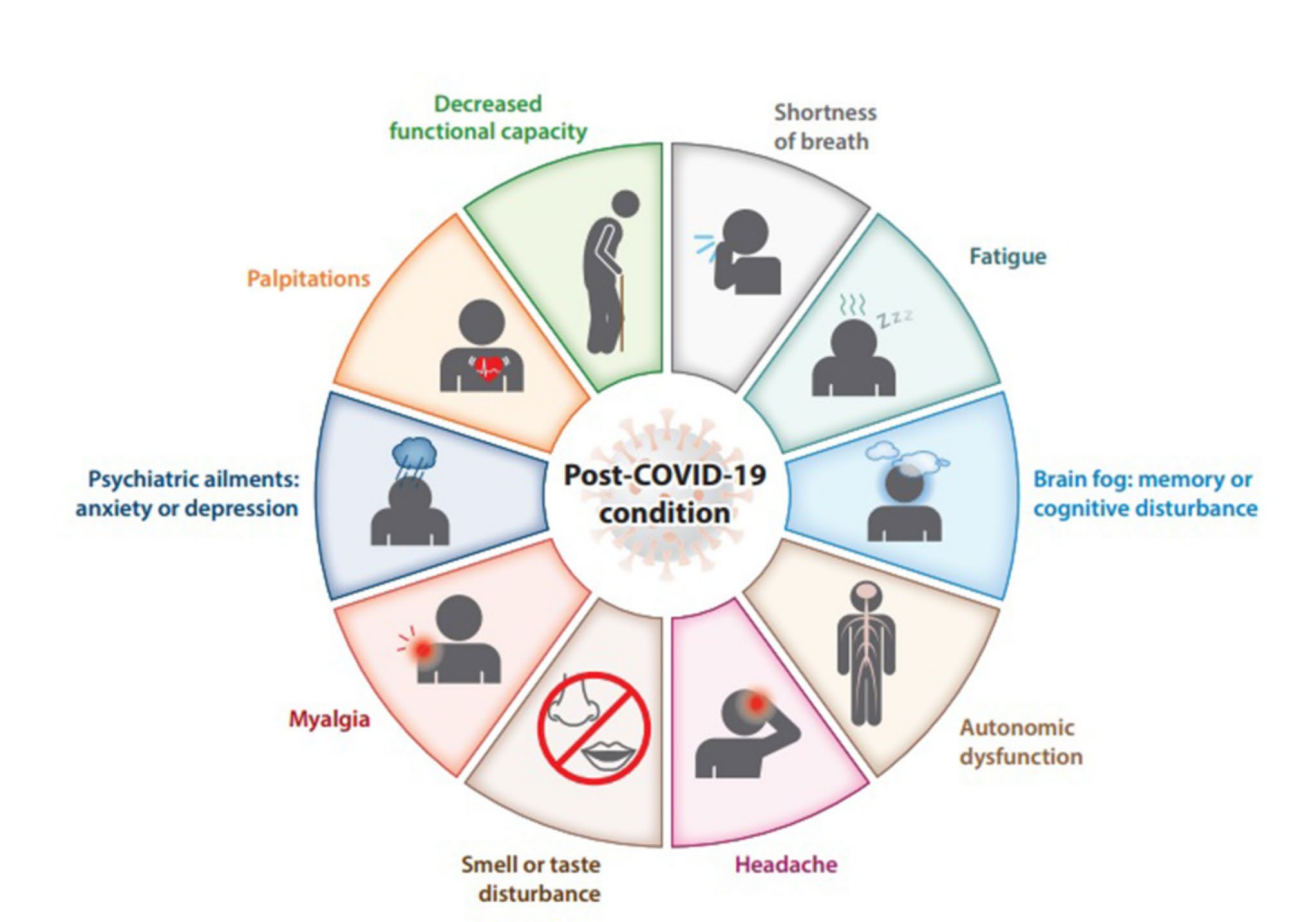
Common manifestations of the post-COVID-19 condition^12^.

Although it may take weeks to recover from the coronavirus disease, some patients’ symptoms continue even after the first infection. It was found that many patients still suffer from long-term complications such as brain fog (which is a cognitive disturbance), fatigue, shortness of breath, myalgia, headache, palpitation, heart failure, and neurological diseases such as stroke and lung diseases as shown in Fig. 1^9–11^. This condition is therefore referred to as post-COVID-19 syndrome^12,13^.

Paracetamol (PAR); N-(4-hydroxyphenyl) acetamide, (Fig.S1(a)) possess analgesic, anti-inflammatory, and antipyretic properties. It blocks cyclooxygenase (especially COX 2), an enzyme involved in the transmission of pain in the brain, acting with extremely precise targeting. It has a different way of action than other painkillers, yet it nevertheless relieves pain throughout the body^14^.

Dexketoprofen Trometamol (DEX), chemically known as (2 S)-2-[3-(benzoyl) phenyl] propanoic acid (Fig.S1 (b)). It is the active optical isomer of ketoprofen, used to treat painful musculoskeletal disorders like osteoarthritis and low back pain, post-operative pain, toothache, and dysmenorrhea^14^. This combination alleviates pain in conditions such as severe muscle pain, headache, mild migraine, tooth pain, rheumatoid arthritis, and osteoarthritis^15^.

Rivaroxaban(RIV),5-chloro-N-([(5 S)-2-oxo-3-[4-(3-oxomorpholin-4-yl)phenyl]-1,3-oxazolidin-5-yl]methyl)thiophene-2- carboxamide,((Fig.S1 (c)), is an oxazolidinone based anticoagulant, is a powerful inhibitor of factor X^16^. It is approved to treat deep vein thrombosis (DVT), prevent recurrent DVT and pulmonary embolism (PE) after an acute DVT in adults, and prevent thromboembolism in individuals with atrial fibrillation^17^.

Thus, Paracetamol and Dexketoprofen Trometamol combination are co-administered by relieved COVID-19 patients for treatment of post-COVID-19 symptoms such as myalgia and headache^12^. Moreover, after being discharged from the hospital, patients with COVID-19 are at risk for thrombotic events; however, extended thromboprophylaxis is recommended by the administration of Rivaroxaban for 35 days post-discharge^18^.

The literature reveals several chromatographic^14,19^and spectrophotometric methods^20^for the simultaneous determination of PAR and DEX in their combined dosage form. On the other hand, some spectroscopic^21–23^, chromatographic^24–31^, and electrochemical methods^32^were also reported for the determination of Rivaroxaban either alone or in combination with other drugs. However, there are no published HPLC/UV methods for simultaneously determining PAR, DEX, and RIV in any biological fluid. Consequently, we aimed to develop and validate a green, sensitive, cost-effective chromatographic method for the simultaneous determination of the mentioned co-administered drugs in bulk powder and spiked human plasma. This method successfully detected PAR, DEX, and RIV in biological fluid, making it a more cost-effective alternative to hyphenated GC-MS and LC-MS methods. The greenness of the procedure was evaluated both qualitatively and quantitatively using two commonly employed Green Analytical Chemistry (GAC) evaluation techniques: the Green Analytical Procedure Index (GAPI)^33^and the Analytical GREEnness measure (AGREE)^34^. Moreover, White Analytical Chemistry (WAC) principles^35^ are used to confirm their environmental impact.

## Experimental study

### Instrumentation

HPLC separations were carried out with a Waters 2695 Alliance HPLC system equipped with a Waters 996 photodiode array detector and the column was Kromasil Phenyl (150 mm x 4.6 mm, 5 μm). Sonication of the samples was carried out using Elma Schmidbauer GmbH Sonicator (model S30H, Singen, Germany). Centrifugation of the spiked samples was performed using Sigma Centrifuge (model no. D-37520 Osterode am Harz, Göttingen, Germany).

### Samples

#### Pure samples

Paracetamol certified to be (100.90 ± 1.310) according to reference method^19^was obtained from (Amriya Pharmaceutical Industries, Amriya, Alexandria, Egypt.), Dexketoprofen Trometamol (100.01 ± 1.578)^19^was brought from (Utopia Pharmaceutical Industries, 10th of Ramadan, Sharqia, Egypt), and Rivaroxaban (99.66 ± 1.890)^16^ was obtained from (National Organization of Drug Control and Research Centre (NODCAR), Omraniya, Giza, Egypt). Diclofenac sodium (DCL) was used as an internal standard (I.S.) and was kindly supplied from (Egyptian International Pharmaceutical Industries (EIPICO), 10th of Ramadan, Sharqia, Egypt.

**Reagents**.

- All reagents were of HPLC-grade without any further purification. HPLC grade ethanol, formic acid, acetonitrile, methanol, ethyl acetate, and sodium hydroxide were purchased from Fisher Scientific (USA).
- Water was double distilled by Automatic Water still (Sci Finetech, Seoul, South Korea).
- Fresh human plasma was purchased from Vaccines and Sera Company (VACSERA) (Cairo, Egypt). The study was approved by the Research Ethics Committee of the Faculty of Pharmacy, Cairo University with ethics approval; No: AC(3243).

### Standard solutions

Stock solutions of PAR, DEX, and DCL containing (1 mg/mL) were prepared separately by dissolving 0.025 g of each drug in 25 mL of mobile phase (0.1% formic acid in water and ethanol (50:50 v/v), except for RIV stock solution; it was prepared by dissolving the drug in acetonitrile: water (80:20 v/v). The stock solutions were then further diluted with the mobile phase to obtain working solutions of concentration (0.1 mg/mL).RIV was prepared in this solvent, unlike the other two drugs due to its insolubility in ethanol or methanol.

### Chromatographic conditions

Gradient chromatographic elution was achieved on Kromasil Phenyl column (150 mm x 4.6 mm i.d, particle size 5 μm) as a stationary phase and a mobile phase containing 0.1% formic acid in water (A): ethanol (B), operated as follows: (95:5 v/v) for 1.5 min, then scaling up to (30:70 v/v) from (7.5 to 8.0 min) and finally returns back to initial concentration from (9.0 to 12.0 min). The flow rate was adjusted to 1.5 mL/min at 25ºC. UV detection was carried out at 254.0 nm using a photodiode array detector and the injection volume was 10.0 µL.

### Procedures

#### Calibration curves

Different aliquots from PAR, DEX, RIV, and DCL working standard solutions (0.1 mg/ mL), were accurately transferred into a set of 10-mL volumetric flasks and the volume was completed to mark with the mobile phase, to obtain final concentrations of (3.00–45.00 µg/mL) PAR, (0.50–50.00 µg/mL) DEX, (0.15–20.00 µg/mL) RIV, and 25 µg/mL I.S (DCL). Ten µL of each solution was injected in triplicates to the HPLC and the chromatograms were obtained using the same procedures as mentioned under “Chromatographic conditions”. The calibration curves were constructed by plotting the relative peak area versus the respective concentration and the regression equations were computed.

#### Spiked human plasma samples preparation

Human plasma aliquots of 200.0µL were individually transferred into a set of 15 mL centrifugation tubes containing serial dilutions of PAR, DEX, RIV, and DCL. The samples were vortexed for 60 s followed by the addition of 800.0µL methanol for protein precipitation to obtain final concentrations ranging from (3.0–45.0 µg/mL), (0.5–45.0 µg/mL), and (0.5 to 45.0 µg/mL) of PAR, DEX, and RIV, respectively, in addition to 25 µg/mL I.S (DCL). Then the tubes were then centrifuged for 10 min at 6000 rpm. Each tube’s supernatant was filtered using syringe filters (0.25 μm). Ten µL of each solution was injected in triplicates to the HPLC and the chromatograms were obtained using the same procedures as mentioned under “Chromatographic conditions”.

## Results and discussion

The proposed method showed reasonable results, confirming its accuracy, precision, and selectivity. The approach has various advantages, including minimal solvent usage, great sensitivity, very small injection volume, and a short run time. Moreover, it offered superiority over other reported HPLC-DAD^14,19^and spectrophotometric^20^ methods for the determination of Paracetamol and Dexketoprofen Trometamol in terms of sensitivity as shown in Table S1. These advantages enable this analytical method to be used in pharmacokinetic research so it can be used as a cheaper alternative method to hyphenated GC-MS and LC-MS methods.

### Optimization of the chromatographic conditions

**Fig. 2:**
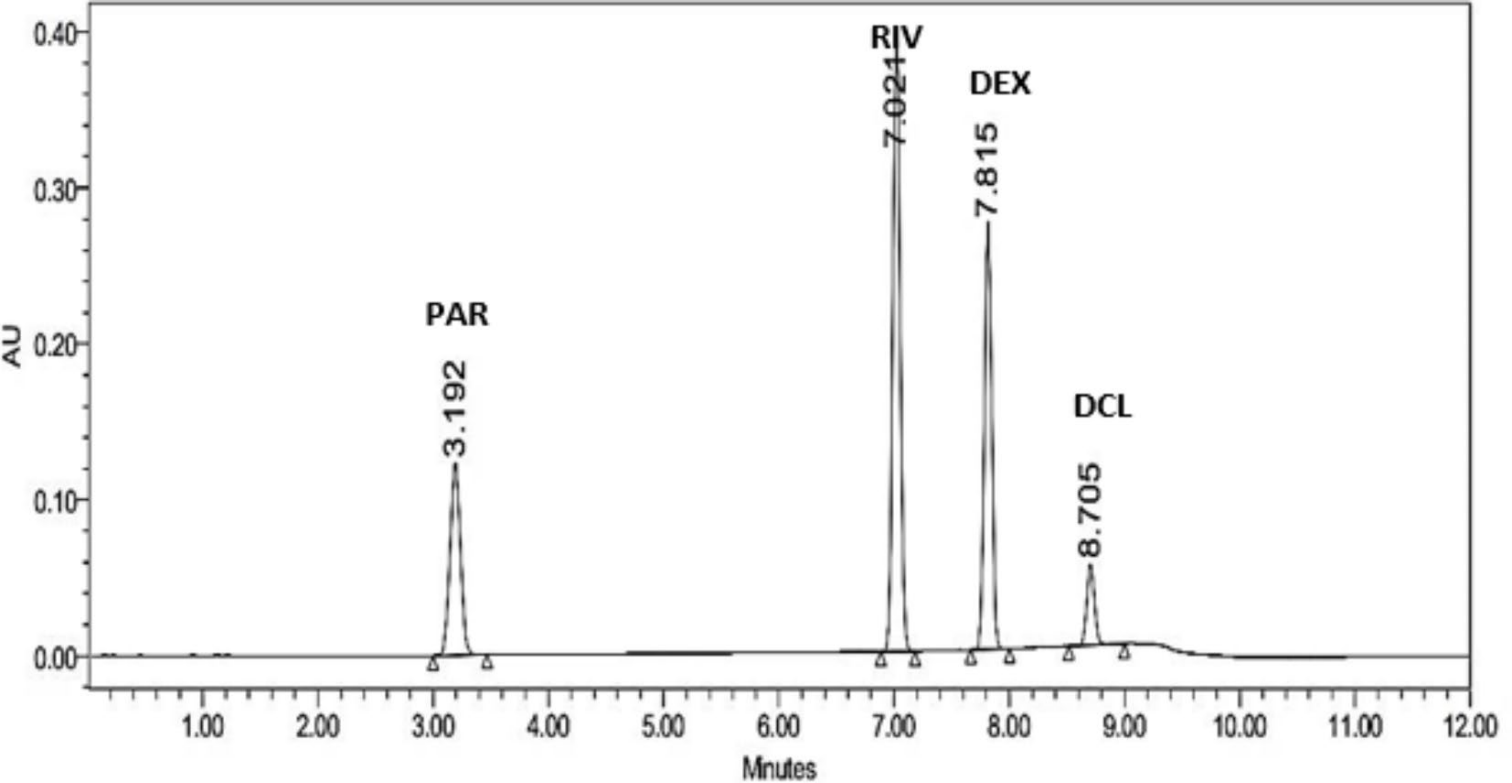
HPLC chromatogram showing simultaneous separation of PAR (200 μg/mL), RIV (200 μg/mL) and DEX (200 μg/mL) in pure form in presence of Diclofenac sodium (25 μg/mL), as an internal standard at 254.0. nm.

Several trials were conducted to obtain the optimal chromatographic conditions for separating the targeted analytes. Various mobile phase solvents, ratios, and column types were tested. Initially, a Kromasil C_18_ (150 mm x 4.6 mm, 5 μm) column with a mobile phase consisting of 0.2% Tri ethylamine adjusted to pH 3.0 by orthophosphoric acid and acetonitrile, in gradient elution mode, was tried. This setup led to overlapping between Rivaroxaban and Dexketoprofen peaks. Switching to a longer column as Kromasil C_18_ (250 mm x 4.6 mm, 5 μm) improved peak sharpness but did not enhance the resolution. Further trials employed different chromatographic conditions and elution modes. For example, Kromasil C_18_ (150 mm x 4.6 mm, 5 μm) column was tested with a mobile phase of (solution (A): 95% of orthophosphoric acid (pH adjusted to 3.0 by NaOH): 5% acetonitrile) while (solution (B): 70% acetonitrile: 30% orthophosphoric acid (pH adjusted to 3.0 by NaOH)) in gradient elution mode. Additionally, an Inerstil C_18_ (150 mm x 4.6 mm, 5 μm) column with a mobile phase of 0.2% tetra butyl ammonium hydrogen sulfate adjusted to pH 6.0 by sodium hydroxide and acetonitrile in ratio (60:40 v/v), in isocratic elution mode. Despite these variations, resolution remained inadequate. Three different internal standards were tried; Caffeine, Prednisolone, and Diclofenac sodium. Caffeine and Prednisolone internal standards were excluded in our study as Caffeine interferes with Paracetamol peak, while Prednisolone interferes with the Rivaroxiban peak. Finally, Diclofenac sodium was the chosen internal standard due to its effective resolution to the other studied drugs. According to the previous trials, the best separation between Paracetamol, Rivaroxaban, Dexketoprofen, and Diclofenac sodium internal standard was obtained using a Kromasil Phenyl column (150 mm x 4.6 mm, 5 μm) as a stationary phase, 0.1% formic acid in water: ethanol which was operated in gradient elution mode as the mobile phase, flow rate 1.5 mL/min, injection volume 10.0 µL and UV detection at 254.0 nm. The obtained chromatogram of the studied drugs is shown in Fig. 2 with retention times 3.1, 7.0, 7.8, and 8.7 min for Paracetamol, Rivaroxaban, and Dexketoprofen Trometamol and Diclofenac sodium, respectively.

### Method validation

The developed method was validated according to ICH guidelines^36^.

## Linearity

The proposed chromatographic method was found to be linear for PAR, DEX, and RIV over the range of (3.00–45.00 µg /mL), (0.50–50.00 µg /mL) and (0.15–20.00 µg /mL), respectively. Calibration curves were constructed by plotting the relative peak area versus the respective concentrations and the assay validation parameters are shown in Table 1.

**Table 1:** Assay validation parameters of the proposed HPLC method for determination of Paracetamol, Dexketoprofen Trometamol and Rivaroxiban.

Parameter	PAR	DEX	RIV
Linearity^a^ (µg/mL)	3.00–45.00	0.5–50.00	0.15-20.00
Slope	0.1268	0.0978	0.1181
Intercept	-0.0257	-0.0014	0.0016
Correlation coefficient(r)	1	1	1
Accuracy^a^ (Mean ± SD)	101.20 ± 0.912	100.45 ± 1.374	100.21 ± 0.850
Intra-day^ab^ (RSD %)	0.318	0.583	0.193
Inter-day^ab^ (RSD %)	0.988	0.860	0.979
Robustness (RSD %)*	0.410 ^c^ 0.325 ^d^	0.061 ^c^ 1.570 ^d^	0.514 ^c^ 0.086 ^d^
LOD(µg/mL)^e^	0.531	0.095	0.047
LOQ(µg/mL)^f^	1.608	0.289	0.143

^a^Mean of three determinations.

^b^Relative standard deviations for concentrations (20.00, 25.00 &35.00 µg/mL) for PAR, (13.00, 20.00 & 30.00 µg/mL) for DEX and (4.00, 10.00 & 20.00 µg/mL) for RIV.

^c^Small deliberate changes in the flow rate (1.5 ± 0.1 mL/min), the tried flow rates were (1.4, 1.5 and 1.6 mL/min).

^d^Small deliberate changes in wavelength of detection (± 2 nm) were made. The tried wavelengths were 252.0, 254.0 and 256.0.

^e^LOD = 3.3 × SD of intercept/slope.

^f^LOQ = 10 × SD of intercept/ slope.

Regression equations were found to be:

Y = 0.1268 x -0.0257 (Correlation coefficient (r) = 1) (For PAR).

Y = 0.09782x -0.0014 (Correlation coefficient (r) = 1) (For DEX).

Y = 0.1181x + 0.0016 (Correlation coefficient (r) = 1) (For RIV).

## Accuracy

The accuracy of the proposed method was evaluated by determination of % recoveries of four different concentrations (10.00, 13.50, 20.00, and 26.00 µg/mL) for PAR, (2.50, 8.00, 13.00, 18.00, and 27.00) for DEX and (0.50, 4.00, 10.00, and 20.00/mL) for RIV using the corresponding regression equation. The method was found to be accurate with good mean % recoveries and standard deviation ranging from (100.21–101.20 ± 0.850–1.374), as shown in Table 1.

## Precision

The interday and intraday precision were evaluated by analyzing three concentrations of the drugs measured in triplicate (20.00, 25.00, and 35.00 µg/mL) for PAR, (13.00, 20.00, and 30.00 µg/mL ) for DEX and (4.00, 10.00, and 20.00 µg/mL) for RIV within the same day to estimate intra-day precision and on three successive days to estimate inter-day precision. The low values of %RSD obtained in both cases within the range of (0.193–0.988) shown in Table 1 suggest excellent precision.

## Selectivity

**Fig. 3:**
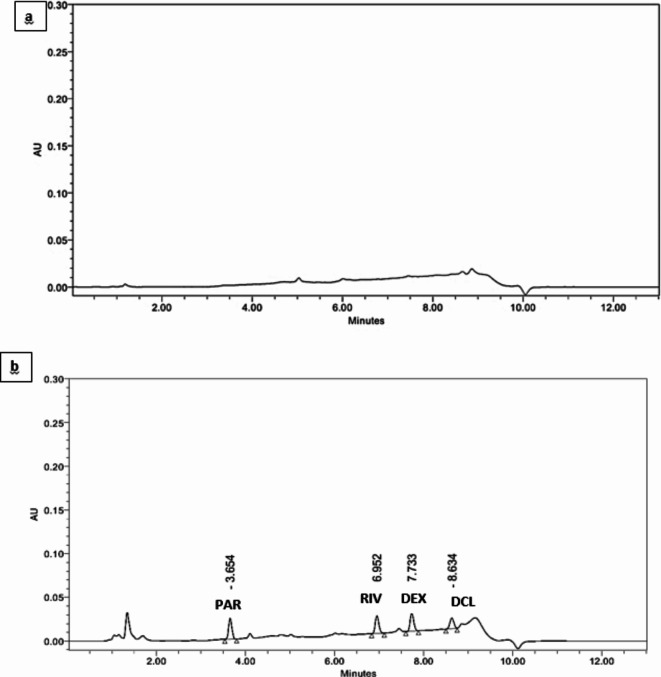
HPLC chromatogram showing blank human plasma extracted with methanol **(a)** and spiked mixture of PAR, RIV and DEX (45 µg/mL each) in presence of Diclofenac sodium (25 µg/mL), as an internal standard at 254.0 nm **(b).**

The selectivity of the proposed method was tested by analyzing blank samples (an extracted supernatant of deprotenized blank human plasma samples) under the same chromatographic conditions. No peaks were detected in the blank sample as shown in Fig. 3(a), proving no interference and hence the selectivity of the proposed method.

## LOD and LOQ

The LOD and LOQ were calculated using the SD of intercept and the slope of the calibration curves, showing high sensitivity of the proposed method as displayed in Table 1, where LOD ranged from 0.047 to 0.531 µg/mL and LOQ from 0.143 to 1.608 µg/mL.

## Robustness

The suggested method was tested for robustness by examining the impact of small deliberate changes in some chromatographic parameters on the obtained results including flow rate (1.5 ± 0.1 mL/min) and wavelength of detection (± 2 nm). The robustness of the suggested method was checked by calculating % RSD; found to be from 0.061 to 1.570% as shown in Table 1, proving the robustness of the proposed method.

## System suitability

The system suitability parameters should be tested to make sure that the system was correctly functioning during the analysis. Capacity factor (K’), selectivity (α), resolution (Rs), tailing factor (T), and the number of theoretical plates were checked and the obtained results were satisfactory when compared to USP reference values^37^ as presented in Table 2.

**Table 2:** System suitability parameters of the proposed chromatographic method.

Parameters	PAR	DEX		RIV	Reference Value^37^.
Retention time (t_R_)	3.19 ± 0.01	7.02 ± 0.01		7.81 ± 0.01	
Capacity factor ( K´)	1.13	3.68		4.21	1 < K < 10
Resolution (Rs)	2.68		6.66		Rs ≥ 1.50
Tailing factor (T)	1.01	1.04		1.02	0.80–1.20
Selectivity factor (α)	-	3.26		1.14	α > 1.00
Theoretical plates number (N)	5821	53,077		66,656	> 2000

### Optimization of the extraction procedure

Optimization of sample preparation is a necessary step for the determination of the investigated analytes in human plasma as it was challenging to recover all analytes from the plasma simultaneously. Thus, two types of sample preparation procedures were tested, such as protein precipitation with methanol, acetonitrile, methanol: acetonitrile (1:1), or liquid-liquid extraction with ethyl acetate in alkaline media. Protein precipitation using either methanol or acetonitrile as an extraction solvent, gave the best recoveries for the three drugs, with the least matrix effect, yet methanol was further chosen rather than acetonitrile as it has a higher green character; Fig.S2 and Table 3.

**Table 3:** The effect of different extraction solvents on the recovery percentage of Paracetamol, Rivaroxiban and Dexketoprofen Trometamol in spiked human plasma.

Drugs	PAR	DEX	RIV
Solvent used	*Recovery %		
Ethyl acetate	32.80	17.24	25.21
Methanol : Acetonitrile (1:1)	77.95	84.75	74.82
Methanol	102.45	99.41	98.24
Acetonitrile	108.79	98.96	100.75

### Application of spiked human plasma

According to their therapeutic levels, the investigated drugs were simultaneously quantified in spiked human plasma. Since the C_max_of PAR, DEX, and RIV were found to be 13.7, 3.71, and 0.5 µg/mL^38–40^, respectively, the sensitivity of the proposed chromatographic method allowed the determination of PAR, DEX, and RIV in spiked plasma as the linearity ranged from (3.0–45.0 µg/mL), (0.5–45.0 µg/mL), and (0.5–45.0 µg/mL) for PAR, DEX, and RIV, respectively, with a good correlation coefficient.

Regression equations were found to be:

Y = 0.0881 x + 0.1482 (Correlation coefficient (r) = 0.9995) (For PAR).

Y = 0.0737x + 0.3985 (Correlation coefficient (r) = 0.9998) (For DEX).

Y = 0.0917x + 0.0601 (Correlation coefficient (r) = 0.9993) (For RIV).

Results presented in Table 4, showed good percentage recoveries and %RSD < 15%, proving the absence of any matrix effect and that the sample processing approach is effective in removing any potential matrix interference as shown in Fig. 3(b).

**Table 4:** Determination of Paracetamol, Dexketoprofen Trometamol and Rivaroxiban in spiked human plasma.

Concentration in spiked human plasma (µg/mL)	PAR	DEX	RIV
(PAR: DEX: RIV)	*Recovery %	*Recovery %	*Recovery %
(3:3:3)	91.49	95.33	99.67
(10:10:10)	99.50	101.60	100.40
(30:30:30)	98.67	100.60	98.30
Mean ± SD	96.55 ± 4.404	99.18 ± 3.367	99.46 ± 1.066

*Average of three determinations.

### Statistical analysis of results

The statistical comparison of the results obtained by the proposed HPLC-DAD method and reference chromatographic methods for the determination of PAR, DEX^19^, and RIV^16^; were shown in Table S2. Results revealed no statistically significant difference in accuracy and precision between the suggested and published methods, as the computed t- and F- values were lower than the tabulated ones.

### Greenness assessment and whiteness assessment

Green analytical chemistry (GAC) is a concept that demands analytical chemists to consider environmental, health, and safety considerations throughout their work^34^. The growing recognition of the harmful influence of chemicals on the environment has increased the urgency for the chemistry community to implement greener and more sustainable analytical processes. However, adopting the GAC concept as an alternative to old harmful procedures and generating greener ways of analysis is difficult due to the wide range of analytes and methodologies, sample matrices, and analytical requirements for validation that must be taken into consideration^41^.

Some tools have been presented to evaluate the greenness of analytical methodologies. The first and oldest green metric used was NEMI^42^. This qualitative procedure is simple and easy to conduct. The primary drawback was that it does not include and describe all 12 principles of GAC^33,43^. Another methodology was used for evaluating the greenness of the methods termed Analytical-ecoscale^42^. It is a semi-quantitative technique that incorporates more GAC principles than NEMI and it takes into account the quantity and hazards of each solvent individually, however, it lacks information regarding the structure of the hazard, with no explanation of the causes of the negative environmental effect on the analytical procedure^33,43^. Thus, two advanced GAC evaluation techniques GAPI^33^and (AGREE)^34^were employed to assist the qualitative and quantitative greenness of the established HPLC method to ensure its greenness. White Analytical Chemistry (WAC) principles^35^ were also tested using the RGB-12 tool to assess their environmental impact.

#### GAPI tool

**Fig. 4:**
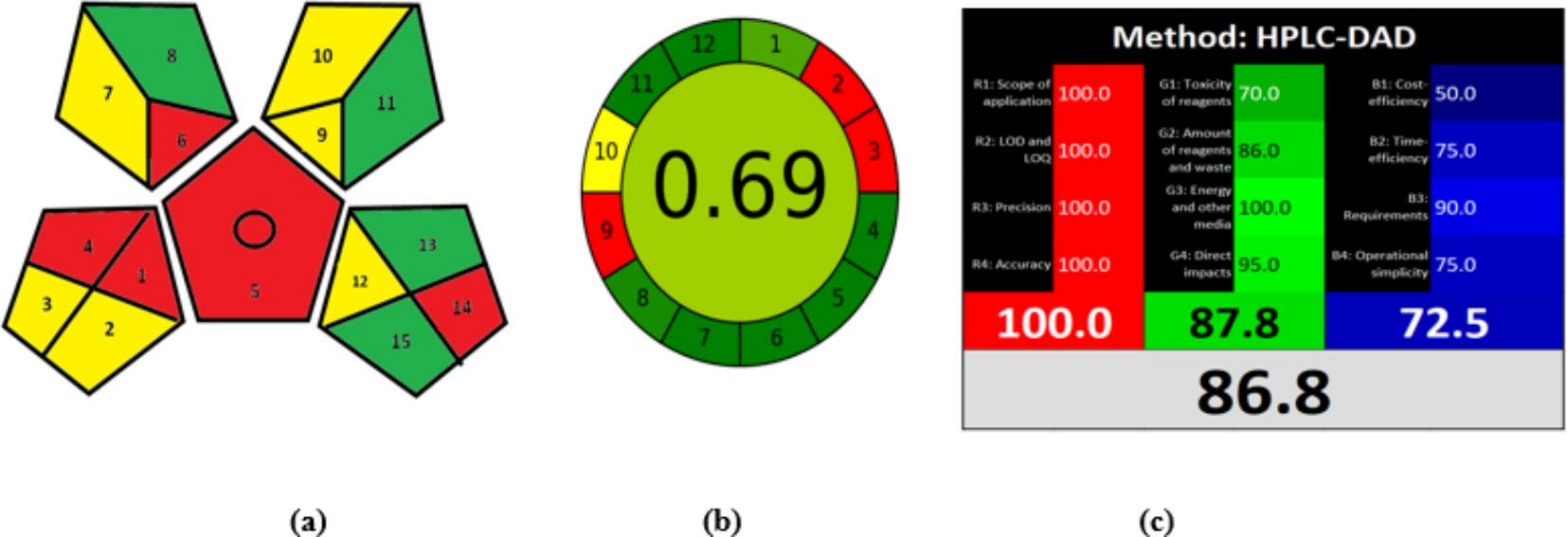
Greenness and whiteness assessment of the proposed method using GAPI**(a)**, AGREE **(b)**,and RGB-12 tools **(c).**

GAPI was first developed by Płotka-Wasylka^33^. GAPI provides a qualitative and quantitative review of the entire analytical method, beginning with sample collection till final analysis. The GAPI pictogram can be green, yellow, or red, with green indicating a safe operation and red indicating a non-eco-friendly procedure. The proposed method applies a green approach, where a green mobile phase of ethanol and water mixture was used and a green extraction technique of protein precipitation using methanol was adopted. As shown in Table S3 and Fig. 4(a), yellow color predominates in most of the pentagrams. Zones (2) and (3) are yellow due to using freezing as a physical preservation technique of the sample and the requirement of transport of plasma samples to the lab. Zones (7),(9), (10), and (12) appear to be yellow too due to using green solvent as methanol in the extraction technique, using the volume of solvent ranging from (10-100mL) and using ethanol in the mobile phase which is considered according to NFPA moderately toxic (NFPA scale = 2). HPLC uses energy of ≤ 1.5 kWh per sample. Five red regions were found, zones (1) and (4) which are due to using an off-line sample collection technique and storage under special conditions; ( freezing). Zones (5), (6), and (14) are red due to the presence of an extraction step and macro extraction technique was applied using methanol and the amount of waste is > 10 mL.

#### AGREE tool

The recently introduced AGREE tool is a calculator that uses a free software to provide a quick, simple, and complete assessment of the green character^34^. It is presented as a final score in the center of the pictogram based on fulfilling the 12 green analytical chemistry criteria. The software automatically provides a pictogram with 12 segments. The width of each segment varies according to its significance. Each segment has a distinct color scale that ranges from green to red. In AGREE, the ultimate score that measures the degree of greenness of the approach is a fraction of one. The outcomes vary from zero to one. When it is closer to one, it suggests more green, and when it is close to zero, it indicates insufficient green. As presented in Table S3 and Fig. 4(b), the final score for the developed method is 0.69. This indicates a good greenness profile for the proposed method.

#### RGB 12 algorithms

Sustainable analytical techniques take into account not only their environmental impact, but also their economic cost, validity, and effectiveness. The White Analytical Chemistry method (WAC) is a useful tool for examining the sustainability of analytical procedures^35^. As stated by White Analytical Chemistry (WAC), RGB 12 consists of three criteria for assessment (red, green, and blue). Each colour describes a certain aspect. Red; (quality of the method and analytical efficiency), green; (the environmental friendliness and safety aspects), and Blue; ( practical and economic aspects). Every criterion in the green, red, and blue principles is evaluated on a scale of 0 to 100. A score closer to 100 indicates a white method, signifying a complete and suitable technique for the application. The suggested chromatographic method demonstrated an outstanding balance between the three characteristics of WAC, scoring an average white score of 86.8 as illustrated in Table S4 and Fig. 4(c). Thus according to WAC evaluation, all principles were equally crucial in maintaining the concept of sustainability.

#### RGB 12 algorithm Vs. AGREE

During the greenness evaluation of the proposed method, the findings obtained from RGB 12 and AGREE concerning the WAC and GAC principles, respectively, are mainly equivalent. However, one must be alert to the variances in criteria and evaluation procedures. Comparing RGB 12 algorithm with AGREE, RGB 12 algorithm showed several advantages over AGREE as it took into consideration the accuracy, LOD, and cost-effectiveness of the proposed method. (i.e. it published real experimental data). Moreover, it introduces new ideas such as genetically modified organisms (GMOs) or animal use in experiments. RGB 12 algorithm also considers 3 criteria together, red, green, and blue which represent the analytical performance, safety considerations, and financial aspects ; respectively. However, this whiteness assessment method has the disadvantage of being subjective as the score is based on the researcher’s ethical responsibility. On the other hand, in AGREE more parameters are taken into consideration. AGREE defines the derivatization step separately and the weight for each criterion. Thus, assessing the greenness using GAC and WAC tools enabled us to get a full image of the green and sustainable character of the proposed method^44,45^. Therefore, it is desirable to employ more than one assessment tool for better evaluation of the green character of the developed analytical procedures^46–48^.

### Conclusion

An HPLC-PDA method was developed and validated for simultaneous determination of co-administered drugs used for the treatment of post-COVID-19 conditions namely PAR, DEX, and RIV in bulk powder, and spiked human plasma. To the best of our knowledge; it is the first method that determines these drugs in a biological sample. The proposed method was validated according to ICH guidelines. This method leverages a green and economical sample preparation approach characterized by high sensitivity and efficient analyte recovery of the cited drugs from the spiked plasma without peak interference from the biological matrix and with a short run time. Owing to the simplicity and successfulness of the proposed method in determining the proposed drugs in spiked human plasma it is applicable for quality control laboratories, bioequivalence and bioavailability centers. The future research plan is the application of the suggested HPLC-DAD method for quantification of co-administered PAR, DEX, and RIV in healthy Egyptian volunteers who were given Infen-P^®^ ( 325.0 mg of PAR & 25.0 mg of DEX) and Xalerto^®^ (20.0 mg of RIV) at the same time. Moreover, calculating the pharmacokinetic parameters as C_max_ (ng/mL), t_max_ (h), t_1/2_ (h), AUC_0–t_, AUC_0−∞_ (ng.h/mL) and elimination rate constant; K_el_ (h^−1^).

## Electronic supplementary material

Below is the link to the electronic supplementary material.

Supplementary Material 1

## Data Availability

Data will be made available on request. The request for materials should be addressed to P.M (pmedhatn1@gmail.com).
