# Supplementary material for: A sustainable and green HPLC-PDA technique for the simultaneous estimation of Post-COVID-19 syndrome co-administered drugs with greenness and whiteness assessment
Source: Sci Rep. 2024 Oct 31;14:26222. doi: 10.1038/s41598-024-75216-4 (PMC11528028; doi:10.1038/s41598-024-75216-4)
Supplement: Supplementary file 1 — Supplementary Material 1 [file 41598_2024_75216_MOESM1_ESM.docx]

**Supplementry data of**

**A sustainable and green HPLC-PDA technique for the simultaneous estimation of Post-COVID-19 syndrome co-administered drugs with greenness and whiteness assessment.**

Passant M. Medhat ^1^, Manal Mohamed Fouad ^1,2^, Hany H. Monir ^3,^ Nermine S. Ghoniem*^3^

^1^ Analytical Chemistry Department, Faculty of Pharmacy, October University for Modern Sciences and Arts (MSA), 11787 6th October City, Egypt.

^2^ Analytical Chemistry Department, Faculty of Pharmacy (Girls), Al-Azhar University, Nasr City, Cairo Egypt.

^3^ Pharmaceutical Analytical Chemistry Department, Faculty of Pharmacy, Cairo University, Kasr El-Aini Street, Cairo ET-11562.

***Corresponding author**: Nermine S. Ghoniem, present address: Pharmaceutical Analytical Chemistry Department, Faculty of Pharmacy, Cairo University, Kasr El-Aini Street, Cairo, Egypt ET-11562.

E-mail address: nermine.ghoniem@pharma.cu.edu.eg

**b**

**a**

**c**

**Fig.S1:** Chemical structure of (a) Paracetamol (b) Dexketoprofen Trometamol (c) Rivaroxaban**.**

**Fig.S2:** Extraction recovery of 25 µg/mL of each of Paracetamol (PAR), Rivaroxaban (RIV) and Dexketoprofen trometamol (DEX) in human plasma using different extraction solvents.

| **Technique** | **Concentration range** | **LOD** | **Run time** | **Application** | **Reference** |
| --- | --- | --- | --- | --- | --- |
| **Proposed HPLC-DAD** | PAR:3-45 µg/mL  DEX: 0.5-50 µg/mL  RIV:0.15-20 µg/mL | PAR:0.531μg/mL  DEX:0.095 μg/mL  RIV:0.047 μg/mL | 12 min | Spiked human plasma | - |
| **HPLC-DAD** | PAR:3-8 µg/mL  DEX: 5-10 µg/mL | PAR:0.05μg/mL  DEX:2 μg/mL | 12 min | Bulk and combined dosage form. | [[1](#_ENREF_1)] |
| **HPLC-DAD** | PAR:50-150 µg/mL  DEX:50-150 µg/mL | PAR:0.15 μg/mL  DEX:2.3 μg/mL | 12 min | Tablet dosage form | [[2](#_ENREF_2)] |
| **TLC** | PAR:25-150ng/spot  DEX:100-600ng/spot | PAR:15 ng/spot  DEX:80 ng /spot | - | Tablet dosage form | [[3](#_ENREF_3)] |
| **Spectrophotometric method** | PAR: 8-28 µg/mL  DEX: 0.4-1.4µg/mL | PAR:0.26μg/mL  DEX:0.02 μg/mL  **For method I** | - | Tablet dosage form | [[4](#_ENREF_4)] |
|  |  | PAR:0.28μg/mL  DEX:0.02 μg/mL  **For method II** |  |  |  |

**Table S1.** Comparison of the proposed HPLC-DAD method in this work with other reported methods for simultaneous determination of Paracetamol and Dexketoprofen Trometamol from literature.

**Table 5.**Greenness and whiteness assessment of the proposed method using GAPI, AGREE and RGB 12 tools.

Table S2.Statistical comparison of the proposed method and the reference method of Paracetamol, Dexketoprofen Trometamol and Rivaroxiban in their pure powdered form.

| **Parameters** | **Paracetamol (PAR)** | | |
| --- | --- | --- | --- |
|  | **HPLC-PDA method** | **Reference Method[**[**2**](#_ENREF_2)**] ^a^** | |
| **Mean** | 101.20 | 100.90 | |
| **SD** | 0.912 | 1.310 | |
| **n** | 4 | 5 | |
| **Variance** | 0.832 | 1.717 | |
| **t-test** | 0.386 (2.365) ***** |  | |
| **F-value** | 2.064 (9.117) ***** |  |  |
| **Parameters** | **Dexketoprofen Trometamol (DEX)** | | |
|  | **HPLC-PDA method** | | **Reference Method[**[**2**](#_ENREF_2)**] ^a^** |
| **Mean** | 100.45 | | 100.01 |
| **SD** | 1.374 | | 1.578 |
| **n** | 5 | | 5 |
| **Variance** | 1.889 | | 2.490 |
| **t-test** | 0.470(2.306) ***** | |  |
| **F-value** | 1.318(6.388) ***** | |  |
|  | **Rivaroxaban (RIV)** | | **Reference Method[**[**5**](#_ENREF_5)**] ^b^** |
| **Mean** | 100.21 | | 99.66 |
| **SD** | 0.850 | | 1.890 |
| **n** | 4 | | 5 |
| **Variance** | 0.723 | | 3.573 |
| **t-test** | 0.528 (2.365) ***** | |  |
| **F-value** | 4.943 (9.117) ***** | |  |

***** Figures in parenthesis are the corresponding theoretical t and F values at (p=0.05)

**^a^** HPLC method for determination of PAR and DEX using Hypersil BDS, C_18_ (250 mm × 4.6 mm x 5μm) column, and mobile phase system of 0.01M Potassium dihydrogen phosphate: acetonitrile (75:25 v/v) pH 6.0 adjusted with triethylamine.

**^b^** HPLC method for determination of RIVA using Phenomenex Luna C_18_ (250 mm × 4.6 mm x 5μm) column, and mobile phase system of acetonitrile : water (55:45 v/v) mixture.

| **Category** | **Proposed Method** |
| --- | --- |
| **Sample Collection (1)** | offline |
| **Sample Preservation (2)** | Physical preservation |
| **Sample Transport (3)** | required |
| **Sample Storage (4)** | Under special conditions |
| **Type of method: direct or indirect (5)** | Extraction required |
| **Scale of extraction (6)** | Macroextraction |
| **Solvents/reagents used (7)** | Green solvent |
| **Additional treatments (8)** | None |
| **Amount (9)** | 10-100 mL  (10-100 g) |
| **Health hazard (10)** | Ethanol: NFPA Health hazard score 2  Formic acid: NFPA Health hazard score 3  Methanol: NFPA health hazard score 1 |
| **Safety hazard (11)** | Ethanol: NFPA flammability score 3 , instabitity score 0  Formic acid: NFPA flammability score 2 , instabitity score 0  Methanol : NFPA flammability score 3 , instabitity score 0 |
| **Energy (12)** | ≤1.5 kWh/ sample |
| **Occupational hazard (13)** | Hermetic sealing of analytical process (No vapours) |
| **Waste (14)** | >10mL |
| **Waste treatment (15)** | Recycling |
| **QUANTIFICATION** | Yes |

**Table S3.** Green Analytical Procedure Index (GAPI) and Analytical GREEnness Metric Approach (AGREE) parameters for our proposed HPLC-PDA method

**Table S4.** Evaluation of whiteness assessment of the suggested HPLC-PDA method using RGB-12 algorithms.

| **RED PRINCIPLES (analytical performance)** |  |  | **R1: Scope of application** | **R2: LOD and LOQ** | | | **R3: Precision** | | | **R4: Accuracy** | | |
| --- | --- | --- | --- | --- | --- | --- | --- | --- | --- | --- | --- | --- |
|  | **Method number** | **Method name** | 0-100 | **LOD** | **LOQ** | 0-100 | **RSD% (repeatability)** | **RSD% (reproducibility)** | 0-100 | **Relative error (%)** | **Recovery (%)** | 0-100 |
|  | **1** | **HPLC-DAD** | 100 | 0.224 | 0.68 | 100 | 0.364 | 0.942 | 100 | 1.044 | 100.62 | 100 |
|  | **2** |  | 0 |  |  | 0 |  |  | 0 |  |  | 0 |
|  | **3** |  | 0 |  |  | 0 |  |  | 0 |  |  | 0 |
|  | **4** |  | 0 |  |  | 0 |  |  | 0 |  |  | 0 |

| **GREEN PRINCIPLES (green chemistry)** |  |  | **G1: Toxicity of reagents (impact and biodegradation)** | **G2: Amount of reagents and waste** | | | | **G3: Consumption of energy and other media** | **G4: Direct impacts (safety, use of animals and GMOs)** | | | |
| --- | --- | --- | --- | --- | --- | --- | --- | --- | --- | --- | --- | --- |
|  | **Method number** | **Method name** | **Total number of pictograms** | 0-100 | **Reagent consumption** | **Waste production** | 0-100 | 1-100 | **Occupational hazards** | **Safety of users**  **(0-100)** | **Use of animals**  **(0 if no, 1 if yes)** | **Use of GMO (0 if no, 1 if yes)** |
|  | **1** | **HPLC-DAD** | 8 | 70 | 24mL | 120 mL | 86 | 100 | 0 | 85 | 0 | 0 |
|  | **2** |  |  | 0 |  |  | 0 | 0 |  | 0 | 0 | 0 |
|  | **3** |  |  | 0 |  |  | 0 | 0 |  | 0 | 0 | 1 |
|  | **4** |  |  | 0 |  |  | 0 | 0 |  | 0 | 1 | 1 |

| **BLUE PRINCIPLES (practical side)** |  |  | **B1: Cost-efficiency** | | **B2: Time-efficiency** | | **B3: Requirements** | | **B4: Operational simplicity** | | | | |
| --- | --- | --- | --- | --- | --- | --- | --- | --- | --- | --- | --- | --- | --- |
|  | **Method number** | **Method name** | **Total**  **cost** | 0-100 | **Speed of analysis** | 0-100 | **Sample consumption** | **Sample consumption**  **(0-100)** | **Other needs: advanced instruments, skills, facilities**  **(0-100)** | **Miniaturization**  **(0-100)** | **Integration and automation**  **(0-100)** | **Portability**  **(0-100)** |  |
|  | **1** | **HPLC-DAD** | intermediate | 50 | 12 mins | 75 | 1 mL | 90 | 90 | 50 | 100 | 75 |  |
|  | **2** |  |  | 0 |  | 0 |  | 0 | 0 | 0 | 0 | 0 |  |
|  | **3** |  |  | 0 |  | 0 |  | 0 | 0 | 0 | 0 | 0 |  |
|  | **4** |  |  | 0 |  | 0 |  | 0 | 0 | 0 | 0 | 0 |  |

**References:**

[1] T.S. Mulla, J.R. Rao, S.S. Yadav, V.V. Bharekar, M.P. Rajput, Development and validation of HPLC method for simultaneous quantitation of paracetamol and dexketoprofen trometamol in bulk drug and formulation, Pharmacie Globale, 7 (2011) 1-4.

[2] D. Pokharkar, R. Korhale, S. Jadhav, N. Birdar, D. Puri, P. Wani, Stability indicating RP-HPLC-PDA method for simultaneous determination of dexketoprofen trometamol and paracetamol from tablet dosage form, Der Pharmacia Letter, 3 (2011) 49-57.

[3] J.R. Rao, T.S. Mulla, V.V. Bharekar, S.S. Yadav, M.P. Rajput, Simultaneous HPTLC Determination of Paracetamol and Dexketoprofen trometamol in pharmaceutical dosage form, Der Pharma Chemica, 3 (2011) 32-38.

[4] L. Kothapalli, A. Karape, A. Thomas, R. Nanda, P. Gaidhani, M. Choudhari, Simultaneous spectrophotometric estimation of paracetamol and dexketoprofen trometamol in pharmaceutical dosage form, Der Pharm, Der Pharma Chemica, 3 (2011) 365-371.

[5] M. Çelebier, T. Reçber, E. Koçak, S. Altınöz, S. Kır, Determination of rivaroxaban in human plasma by solid-phase extraction–high performance liquid chromatography, J. Chromatogr. Sci., 54 (2016) 216-220.
